# Electrical Conduction Mechanism of Mg-Doped ZrO_2_ Thin Films

**DOI:** 10.3390/ma17153652

**Published:** 2024-07-24

**Authors:** Diana Mardare, Mariana Frenti, Carmen Mita, Nicoleta Cornei, Georgiana Bulai, Marius Dobromir, Alexandr Doroshkevich, Abdullah Yildiz

**Affiliations:** 1 Faculty of Physics, “Alexandru Ioan Cuza” University of Iasi, 11 Carol I Blvd., 700506 Iasi, Romania; dianam@uaic.ro (D.M.); marianafrenti2009@gmail.com (M.F.); 2 Faculty of Chemistry, “Alexandru Ioan Cuza” University of Iasi, 11 Carol I Blvd., 700506 Iasi, Romania; ncornei@uaic.ro; 3 Integrated Centre for Environmental Science, Studies in the North-East Development Region—CERNESIM, “Alexandru Ioan Cuza” University of Iasi, 11 Carol I Blvd., 700506 Iasi, Romania; georgiana.bulai@uaic.ro; 4 Research Center on Advanced Materials and Technologies, Department of Exact and Natural Sciences, Institute of Interdisciplinary Research, “Alexandru Ioan Cuza” University of Iasi, 700506 Iasi, Romania; marius.dobromir@uaic.ro; 5 Joint Institute for Nuclear Research, Str. Joliot-Curie, 6, 141980 Dubna, Russia; doroh@jinr.ru; 6 Faculty of Engineering and Natural Sciences, Department of Energy Systems Engineering, Ankara Yıldırım Beyazıt University, 06010 Ankara, Turkey

**Keywords:** Mg doped ZrO_2_, thin films, XPS, electrical conductivity, Meyer-Neldel rule

## Abstract

Amorphous ZrO_2_ thin films with increasing Mg content were deposited on quartz substrates, by dip coating method. The films are transparent in the visible domain and absorbent in UV, with an optical band gap that decreases with the increase of Mg content, from 5.42 eV to 4.12 eV. The temperature dependent conductivity measurements showed typical semiconductor comportment. The decrease of the electrical conductivity by Mg doping was related to the increase of the OH groups (37% to 63%) as seen from X-ray Photoelectron Spectroscopy. It was found out that the electrical conductivity obeys the Meyer-Neldel rule. This rule, previously reported for different disordered material systems is obtained for ZrO_2_ for the first time in the literature. Exploring novel aspects of Mg-doped ZrO_2_, the present study underscores the origin of the Meyer-Neldel rule explained by the small-polaron hopping model in the non-adiabatic hopping regime. Determination of the presence of such a conduction mechanism in the samples hold promise for comprehending the important aspects, which might be a concern in developing various devices based on Mg-doped ZrO_2_.

## 1. Introduction

Zirconium dioxide is a transition metal oxide that is evidenced through some special properties like: high thermal stability, high corrosion resistance, high chemical and mechanical stability, low thermal expansion coefficient, high electrical resistivity, high transparency in visible and near infrared. These properties make it suitable in different applications such as: ceramic material, piezoelectric material, magnetic materials, high dielectric material for large-scale integrated circuits, catalytic material etc. [1,2]. Zirconia can crystallize in three stable structures: monoclinic, tetragonal, and cubic. While the monoclinic phase is stable at room temperature, the tetragonal and cubic phases become stable at temperatures above 1473 °C and 2673 °C, respectively. Different dopants (Sc, Y, Ca, Mg) can stabilize the high temperature cubic phase at room temperature [3]. But in the absence of an appropriate stabilization, this phase transforms to the tetragonal and then to the stable monoclinic phase, the transformations being attended with an increase in volume [4]. Magnesium is a divalent cation, having a proper size for the host lattice; both Mg and Zr have an ionic radius of 0.072 nm (six coordinated) and can induce the above-mentioned stabilization by direct substitution [5]. Having a lower valence (Mg^2+^) than zirconium (Zr^4+^), the lattice neutrality is maintained by the appearance of the oxygen vacancies [6].

Researches made on the electrical conductivities of bulk zirconia compared to the ones of zirconia thin films deposited by different methods, and doped with different cations, conduct to inconsistencies that rise the necessity of further investigations [7,8,9,10].

This paper presents the properties (structure, surface elemental composition, optical band gap, electrical conductivity) of the undoped and Mg-doped ZrO_2_ thin films (for different Mg concentrations) deposited by dip coating method and explains the mechanism of the electrical conduction in these films, related to the Meyer-Neldel rule (MNR).

## 2. Materials and Methods

Zirconia thin films with an increased magnesium content (1, 2, 3% mol) have been obtained by the dip coating method on quartz substrates (2 cm × 2 cm). The films were suggestively denoted as: M1, M2, and M3, the notation M0 corresponding to the undoped one. Before deposition, the substrates were cleaned with acetone, immersed for 2 h in hydrochloric acid solution (20%), then washed with water and dried in air. Each film was obtained by dipping the substrate in the corresponding solution and then picked up with a speed of 7 mm/s. The procedure was repeated 3 times; after each immersion the obtained films were dried at a temperature of 80 °C for 2 h, and then thermally treated at 300 °C for 5 min. Finally, the obtained layers were densified by thermal treatment at 500 °C, for 30 min. To prepare the immersion solutions, first, solutions of ZrCl_4_ (Aldrich, Wyoming, IL, USA—99.5% purity) and Mg(NO_3_)_2_ were obtained according to the amounts expressed in moles fr, om Table 1. By mixing them, and adding an appropriate amount of distilled water, a volume of 50 mL was obtained from each solution. The corresponding final solutions were obtained by adding to the solutions of ZrCl_4_ + Mg(NO_3_)_2_ mixtures, some amounts of ethylene glycol (moles) corresponding to the ratio mol_metal cation ratio (Zr + Mg)_/mol_ethylene glycol_ = 1/2.

The structure of the films was studied by X-ray diffraction (XRD), using a diffractometer Shimadzu 6000 (Shimadzu, Kyoto, Japan), in θ–2θ geometry (40 kV, 30 mA, CuKα radiation: 1.540591 Å).

The thickness of the films, determined with a Dektak XT stylus profilometer (Bruker, Wissembourg, France), was found to be 200 nm.

The X-ray photoelectron spectroscopy (XPS) technique was used to obtain the elemental composition in the films’ surface. The XPS spectra were recorded with a Physical Electronics PHI 5000 VersaProbe instrument (Ulvac-PHI, Inc., Chikasaki, Japan), equipped with a monochromatic Al Kα X-ray radiation (hν = 1486.7 eV).

The values of the optical band gap of the studied films were derived from the optical transmittance spectra, measured with a Camspec 501M spectrophotometer (Camspec, Garforth, United Kingdom, (190–1100 nm) having an integrated sphere coated with BaSO_4_ against quartz as blank. Assuming that the direct allowed transition dominates over the optical absorption in ZrO_2_ [11], the values of the films’ optical band gap can be obtained from these spectra.

A home-built device was used to investigate the electrical conduction mechanism in the undoped and Mg-doped ZrO_2_ thin films. The device consists in a cubic glass precinct containing a heater, on which the thin film is placed, and a ventilation system used to homogenize the gas. The films electrical resistances were measured between two parallel electrodes (silver painted, 1 mm distance). The measurements are performed in air as a function of temperature. A tera ohmmeter (UNI-T UT510 Uni-Trend Technology, Munich, Germany) is used to determine the electrical resistance of the films and a K-probe temperature sensor connected to the TM902C digital temperature measuring device, is placed on the film surface to determine its temperature.

## 3. Results and Discussion

### 3.1. X-ray Diffraction (XRD)

**Figure 1:**
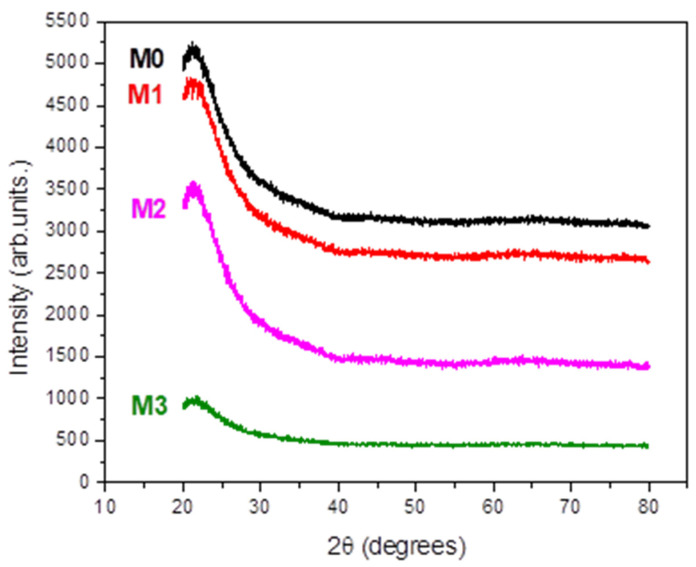
XRD patterns of the studied films.

X-ray Diffraction (XRD) studies revealed that all the investigated films are amorphous (Figure 1).

### 3.2. X-ray Photoelectron Spectroscopy

The XPS measurements were carried out in order to reveal the elemental composition of the films surface. Table 2 shows the binding energy and area of the peaks obtained after the gaussian deconvolution of XPS high-resolution spectra of zirconium, magnesium and oxygen corresponding to *Zr 3d*, *Mg 3p_3/2_* and *O 1s* curves, respectively.

**Figure 2:**
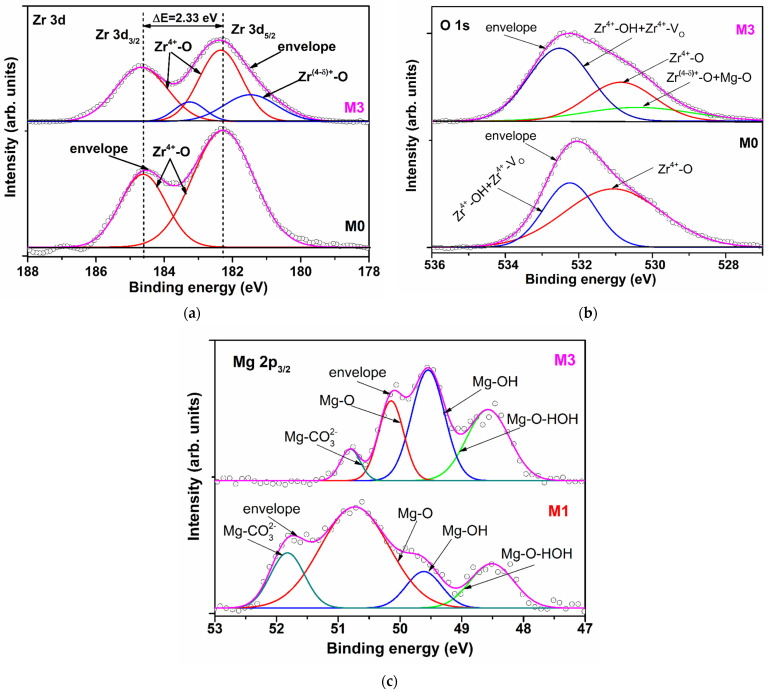
High-resolution spectra and peak fitting of (**a**) Zr 3d and (**b**) O 1s for M0 and M3 and (**c**) Mg 2p_3/2_ for M1 and M3.

The Zr 3d XPS high-resolution spectra of M0 sample was split in two peaks located at 182.28 eV and 184.61 eV, assigned to Zr 3d_5/2_ and Zr 3d_3/2_, respectively (Figure 2a) [12]. For the Mg-doped films, besides the main peaks, lower binding energies were identified (Table 2, Figure 2a). This split of peaks can be due to the shake-up state of Zr 3d orbital determined by the increase of zirconium cation electronic density in the certain direction as a consequence of the increase of disorder degree in the structure of the film surface. The disorder is induced by the random substitution of Zr^4+^ by a cation with half ionic charge (Mg^2+^), but with the same radii (r = 0.072 nm for the coordination number C.N. = 6 and r = 0.066 nm for C.N. = 5 [13]), which may create anionic disorder expressed by increasing of non-equivalent oxygen environments and oxygen vacancies, respectively. The incorporation of magnesium cations in the zirconia matrix (M0) induced a shift of the Zr 3d_5/2_ and Zr 3d_3/2_ main peaks binding energies to higher values for the film M1, and a shift to lower values for M2, and M3. So, the decrease is from 182.65 eV (M1) to 182.12 eV (M3) and from 184.97 eV (M1) to 184.45 eV (M3), respectively (Table 2). The same tendency was observed for the lower binding energy peaks Zr 3d_5/2_: from 181.58 eV (M1) to 181.27 eV (M3). These progressive decrease of positively core shell level of Zr^4+^ (design as Zr^(4-δ)+^ in Figure 2a) with the doping level can be attributed to the temporarily capturing of one electron and therefore an increase of the compensation effect of negatively charge reduction [14] and/or to the presence of oxygen species associated with smaller charge negatively contribution (HO^−^,O_2_^−^) [15,16]. The spin-orbit splitting of Zr 3d_5/2_ and Zr 3d_3/2_ core levels around 2.30 eV suggests that the maximum oxidation state of zirconium is conserved but with an enhanced contribution of oxygen species with lower negative charge. The above observations are confirmed by the increase of Zr^(4-δ)+^-O bonds total contribution from 4.52% for M1 to 24.45% for M3.

The binding energy components of O 1s for the under-study films are presented in Table 2 and for M0 and M3 are depicted in Figure 2b. They consist in two peaks for M0 and M1 and three peaks for M2 and M3. The peaks located at 531.06 eV (M0), 530.44 eV (M1), 530.65 eV (M3) and 530. 86 eV (M3) were assigned to Zr^4+^-O lattice. The peaks centered at 529.62 eV, 529.62 eV (M2) and 530.40 eV (M3) were attributed to Zr^(4-δ)+^-O and Mg^2+^-O bonds [17], having a contribution of about 2%, 3% and 16%, respectively from the total area of O1s peaks. The much higher contribution of Zr^(4-δ)+^-O and Mg^2+^-O bonds could be attributed to an increased concentration of Mg^2+^ on the M3 surface despite the lack of difference between Zr^4+^ and Mg^2+^ atomic radii (for the same C.N.). This will induce the supplementary oxygen vacancies which will favour the water molecules adsorption. The binding energy assigned to Zr-OH and Zr-VO bonds (where VO is oxygen vacancy) (about 532 eV—Table 2) and the contribution of the second O 1s peak for M0 (36.94%) and the third peaks for M1–M3 (49.60%, 52.39% and 63.40% respectively) confirm the above statement. The slightly increase of the binding energy with magnesium doping level can be attributed to an increase of OH groups contribution against VO concentration.

XPS high-resolution spectra of Mg 2p_3/2_ of the films M1 and M2 are larger than for the film M3. The Mg 2p_3/2_ signal can be fitted in four components for all the doped films. The binding energies and the corresponding peak areas are presented in Table 2 and Figure 2c for M1 and M3 films. The peaks located at 48.51 eV (M1), 48.30 eV (M2) and 48.57 eV (M3) we assigned to Mg-O-HOH (water) bonds which have a less covalent character than Mg-OH bonds because the obtained method of under study films didn’t facilitate the reduction of Mg^2+^ to Mg^0^ [18]. The second peaks, centered at 49.61 eV (M1), 49.61 eV (M2) and 49.54 eV (M3), having 10.74%, 26.50% and 38.99% contribution, respectively, correspond to the Mg-OH groups. 1 eV differences between the Mg-O-HOH (water) and Mg-OH bonds confirm the inhomogeneous charging of these two bonds. The cumulative contribution of Mg-O-HOH (water) and Mg-OH bonds show that the most adsorptive centers of the lattice are located on/or next to the manganese atoms.

Higher binding energy of peaks located at 50.73 and 50.82 eV (M1), 50.03 and 50.14 eV (M2), 50.14 and 50.80 eV (M3) were associated with Mg-O and Mg-CO_3_^2−^ bonds, respectively. The presence of CO_3_^2−^ ions are confirmed by the C 1s peaks located at about 286 eV (Table 2). The Mg-O/Mg-CO_3_^2−^ contribution bonds ratio increases with doping level from 3.25 for M1 to 5.63 for M3, which indicates that the CO_2_ quantity adsorbed on the film surfaces don’t dominate adsorption process, suggesting that HO groups are the main factor which influences the conduction properties of the Mg doped zirconia films.

### 3.3. Optical Band Gap

**Figure 3:**
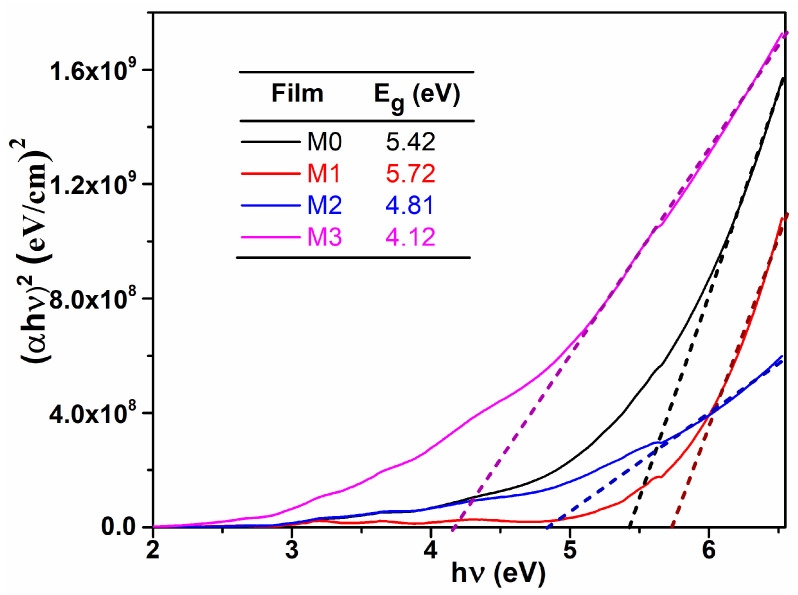
${\left(\alpha h\nu \right)}^{2}$ vs. hν plots; determination of the optical band gap values for the studied films.

While passing through a material, the light intensity decreases due to two phenomena: absorption and scattering. In the energy domain where the fundamental absorption of light is the dominant phenomena, the absorption coefficient (α) at a certain wavelength is given by the relation [19]:

(1) $$\alpha =d^{-1}\ln(1/T),$$

where T is the measured optical transmittance at the same wavelength and d is the thickness of the film. According to the literature [11], we assume that in the neighborhood of the fundamental absorption of ZrO_2_, direct allowed transitions are dominant and the dependence α(*hν*), is given by the relation [2]:

(2) $${\left(\alpha h\nu \right)}^{2}=B(h\nu -E_{g}),$$

where *E_g_* is the optical band gap energy and B is a constant which does not depend on energy. The values of E_g_ were obtained by extrapolating the linear part of the dependence ${\left(\alpha h\nu \right)}^{2}=f(h\nu )$ to ${\left(\alpha h\nu \right)}^{2}=0$. From Figure 3, one can observe that the optical band gap decreases with Mg content, which could be important in some applications, like photocatalysis, were the light from the sun can be efficiently used. These values are consisted with those reported in literature [14], being suggested that Mg dopant determine an alteration of the electronic states between the two allowed bands (conduction band and valence band).

### 3.4. Electrical Conductivity

**Figure 4:**
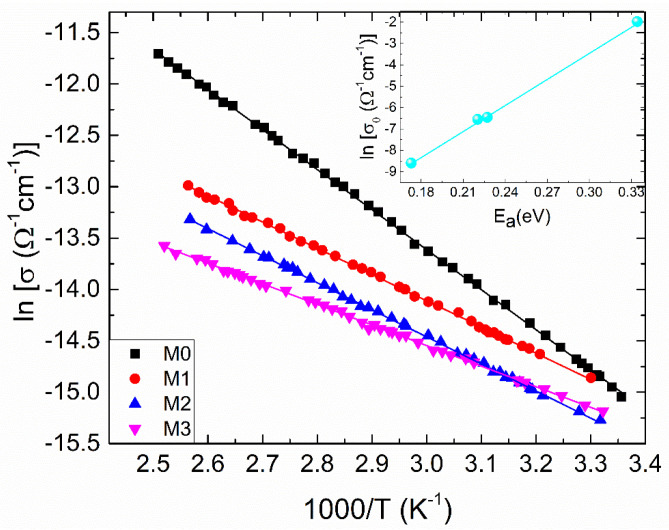
The temperature dependent electrical conductivity for the samples under investigation, plotted as ln (*σ*) versus 1000/T. With respect to Equation (3), solid lines represent the best-fit lines. Preexponential factor (*σ*_0_) and activation energy (E_a_) relationship is shown in the inset; the solid line is the best-fit line with Equation (4).

As presented in Figure 4, temperature-dependent electrical conductivities (*σ*) are assessed to explore the electrical conduction mechanism of zirconia and Mg-doped zirconia films. It can be observed that the dependences show typical semiconductor behavior (d*σ*/d*T* > 0) and the conductivity of ZrO_2_ decreases once Mg dopant enters its lattice. There are different explanations for this decrease, for different experimental conditions: Deng et al. [20] reported the electrical conductivity of ZrO_2_ at temperatures higher than 1000 °C which increases firstly with dopant content, reaches a maximum at a certain value of Mg dopant, and then declines with rising dopant concentration of Mg. They stated that the conductivity for Mg doped ZrO_2_ samples decreases because of the deterioration of the cubic solid solution and the binding between the dopant cation and the oxygen vacancy, which was found to have a substantial impact on the conductivity. Vijayakumar et al. [21] noticed that the variations in charge carrier concentration with Mg dopant in ZrO_2_ samples affects the conductivity. At 18 mol% Mg-ZrO_2_, the conductivity declines compared to the undoped ZrO_2_ sample. Mbae et al. [22] studied the band structure of undoped and Mg-doped ZrO_2_, clearly demonstrating a shift in the Fermi level towards the valence band for the doped sample, with an important role in conductivity. The shift was explained by charge compensation, since a Zr^4+^ ion has been replaced by a dopant with an oxidation state of +2. Yoon et al. [8] attributed the conductivity degrading by Mg doping to the increase in defect association (dopant–vacancy interaction), which reduces the vacancy mobility. Renuka et al. [6] reported that Mg dopant causes a decline in crystal quality of ZrO_2_ with lowering crystallite size. In this case, grain boundaries can become operative, trapping the carries and decreasing the conductivity.

In this paper, the decrease in the electrical conductivity of ZrO_2_ by Mg doping was correlated with the increasing concentration of the hydroxyl groups evidenced from XPS. So, even the oxygen vacancies concentration increases by doping, due to charge compensation, these were filled by the species generated of dissociative chemisorption of ambient atmosphere water molecules (75% relative humidity).

In amorphous semiconductors, the Mott variable-range hopping (VRH) model fits well to explain the conduction process at sufficiently low temperatures, which are typically lower than room temperature [23,24,25]. In the VRH regime, carrier transport is dominated by hopping between defect states that are relatively close to the Fermi level [23,24,25]. On the other hand, the carrier transport can be explained by the Arrhenius model at higher temperatures.

Commonly for a semiconductor, its electrical conductivity, σ, can be thermally activated, being well described by the Arrhenius relation [26] given below:

(3) $$\sigma =\sigma_{o}exp\left(-\frac{E_{a}}{k_{B}T}\right),$$

where σ_o_, E_a_, k_B_ and T denote the pre-exponential factor, thermal activation energy, Boltzmann’s constant and absolute temperature, respectively. The dependence (3) is sustained for the investigated films, since there is a linear dependence of lnσ as a function of inverse temperature (Figure 4). The inset in Figure 4 represents the dependence of ln σ_o_ versus E_a_, parameters determined from the linear fitting. Accordingly, it is quite clear from the figure that there is a linear relationship between these parameters, which can be expressed by [27]:

(4) $$\sigma_{o}=\sigma_{oo}exp\left(\frac{E_{a}}{E_{MN}}\right),$$

The correlation between σ_o_ and E_a_ is called the Meyer-Neldel rule [27]. Here, σ_oo_ and E_MN_ denote the MNR pre-exponential factor and the MNR characteristic energy, respectively.

The origin of MNR, mainly ascribed to the disorder or distortion in the lattice [27,28], still remains not clear. This rule was previously reported for various disordered material systems [28,29,30,31]. For instance, the explanation of the MNR observed in the electrical conductivity of CdS films, given by Hariech et al. in [31], was correlated with the disorder in the films’ matrices and with the contribution of localized states in the electrical transport. Kumar et al. reached to the same conclusion for the investigated glassy alloys Se_75_In_25-x_Pb_x_ thin films [28]. Hariech et al. consider that, even if MNR is attributed to the disorder effect in a material, its origin is connected to the dominant transport mechanism [31]. In Ref [32] Jakson et al. report that MNR could be also a consequence of the multi-trapping transport process. It is worth saying that, as far as we know, the finding of the Meyer-Neldel rule in ZrO_2_ thin films, is obtained here for the first time in the literature.

To elucidate the electrical conduction mechanism in the investigated films, we have carried out a thorough investigation based on MNR. By substituting σ_o_ from Equation (4) into Equation (3), one can find:

(5) $$\sigma =\sigma_{oo}exp\left[\left(\frac{1}{E_{MN}}-\frac{1}{k_{B}T}\right)E_{a}\right],$$

This yields a single crossing point, at a certain temperature, for various values of E_a_. This temperature is known as the MNR temperature (T_MN_), being estimated as:

(6) $$T_{MN}=\frac{E_{MN}}{k_{B}},$$

It is important to observe that at T_MN_, *σ* becomes independent of E_a_. The values of σ_oo_, E_MN_ and T_MN_ are found to be: 1.61 × 10^−7^ (cm)^−1^, 24.62 meV and 285.5 K, respectively. These values fall within the ranges reported for various disordered materials [26,28].

It is evident from the MNR analysis that $T_{MN}=\theta_{D}/2$, where $\theta_{D}$ is the Debye temperature previously reported between 564–575 K for ZrO_2_ [33,34]. Since $T_{MN}=\theta_{D}/2$, one can expect that the small-polaron hopping (SPH) conduction is the dominant conduction mechanism in the investigated samples [35]. Small polarons are quasiparticles made up of a charge carrier and a related lattice deformation. Because of the strong carrier-phonon coupling, carriers can frequently self-trap to form small polarons. Small polaron have substantially detrimental effect on the performance of material in various applications. Small polarons are obviously destructive for photovoltaics since they significantly reduce charge-carrier mobilities, cause charge-carrier self-trapping and carrier recombination [36]. However, small polarons could be highly useful for solid-state lighting and gamma-ray detection [37,38]. Therefore, it should be addressed for Mg-doped ZrO_2_.

The SPH mainly occurs in either adiabatic or nonadiabatic regime. These regimes can be identified by the possibility for the polaron to jump to the adjacent site. This possibility is high and low, for the adiabatic and nonadiabatic regime, respectively.

**Figure 5:**
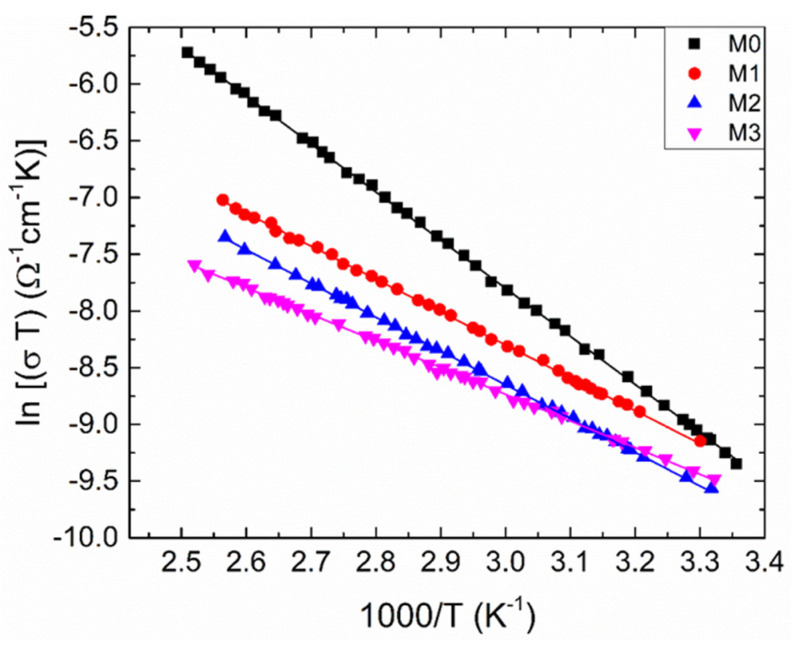
The temperature dependent electrical conductivity for the samples under investigation as ln (σT) versus 1000/T. Solid lines are calculated by utilizing the least-squares technique.

To clarify the nature of the SPH, we have to plot the dependence ln (*σ*T) versus 1000/T (Figure 5), this dependence being well-suited to explain the SPH. Then we can reveal the charge-transfer processes in both the adiabatic and non-adiabatic conditions.

In the frame of the SPH model, the electrical conductivity in the non-adiabatic regime is given as [39]:

(7) $$\sigma ={(\sigma }_{p}/T)exp(-E_{p}/k_{B}T),$$

where σ_p_ is:

(8) $$\sigma_{p}={(v}_{0}/R)e^{2}C(1-C)exp(-2\eta R/k_{B}),$$

In the relations (7) and (8), $\sigma_{p}$ and E_p_ denote the pre-exponential factor and the activation energy, respectively; $v_{0}$ is the optical phonon frequency, being about 10^13^ Hz; C and R stand for the fraction of transition metal ions and the average distance between the ions, respectively. Herein, the value of C can be estimated from the measurement of temperature dependent Seebeck coefficient [40]; η corresponds to the inverse of location length; e is the electron charge; k_B_ is the Boltzmann’s constant. In the adiabatic regime, $exp(-2\eta R/k_{B})$ approaches to 1. We fitted the measured data with Equation (7) (Figure 5). The estimated values of E_p_ are listed in Table 3. Knowing E_p_ values, one can obtain the polaron hopping energy ($E_{h}$). In these equations, $R=2.48r_{p}$. where $r_{p}$ is the polaron radius. The following equations can be considered:

(9) $$\varepsilon_{p}=\frac{e^{2}}{{4E_{p}r}_{p}},$$

and

(10) $$\frac{1}{\varepsilon_{p}}=\frac{1}{\varepsilon_{\infty }}-\frac{1}{\varepsilon_{s}},$$

where $\varepsilon_{p}$ is the effective dielectric constant, $\varepsilon_{\infty }$ (=4.6) and $\varepsilon_{s}$ (=21) are the high and static dielectric constants of ZrO_2_, respectively [41]. Then, we can derive the polaron hopping energy, $E_{h}$, defined as:

(11) $$E_{h}=(\frac{e^{2}}{4\varepsilon_{p}})(\frac{1}{r_{p}}-\frac{1}{R}),$$

We can also calculate the disorder energy (E_d_), defined as:

(12) $$E_{d}=(\frac{0.3e^{2}}{\varepsilon_{s}R}),$$

The energy E_p_ consists of these energies for $T>\theta_{D}/2$, expressed as:

(13) $$E_{p}=E_{h}+\frac{E_{d}}{2},$$

Note that these values tabulated in Table 3 are in a good agreement with those reported in the literature [40,41,42,43,44].

The possibility of polaron hopping is proportional to the transfer integral (J) given as [43]:

(14) $$J=0.67\times hv_{0}{\left(\frac{T}{\theta_{D}}\right)}^{1/4},$$

We calculated the value of J to be as 28.7 meV at 330 K. If the value of J is big enough, adiabatic regime is operative. Otherwise, non-adiabatic regime becomes important. Considering a relationship reflecting how high hopping possibility is, one can distinguish between adiabatic and non-adiabatic regimes. To do so, the value of J should be compared to H expressed by the following relation [45]:

(15) $$H={\left(\frac{2k_{B}TE_{h}}{\pi }\right)}^{1/4}{\left(\frac{hv_{0}}{\pi }\right)}^{1/2},$$

If J > H, hopping takes place in adiabatic regime, while hopping occurs in non-adiabatic regime when J < H. The values of estimated H are given in Table 3. So, one can see that the polaron travels via the non-adiabatic hopping regime in the films under study. To determine the value of the SPH coupling constant ($\gamma_{p}$), which indicates how strong the carrier-phonon interaction is, one can use the relation [39]:

(16) $$\gamma_{p}=\left(\frac{2E_{h}}{hv_{0}}\right),$$

Since $\gamma_{p}$ is higher than 4 for all the samples, it confirms that a strong carrier-phonon interaction happens in the samples and carrier transport is associated with SPH mechanism. Finally, the samples should fulfil the condition of SPH model given below [39]:

(17) $$J<E_{h}/3,$$

The condition is satisfied with all the samples, confirming the applicability of SPH model to the samples.

## 4. Conclusions

In this paper, we have reported on the electrical conduction mechanism of amorphous Mg-doped ZrO_2_ thin films, deposited by dip coating. A detailed XPS analysis was performed. The optical band gap, obtained from optical transmittance spectra, decreases by Mg doping, from 5.42 eV to 4.12 eV. By studying the dependence of the electrical conductivity as a function of temperature, a characteristic semiconductor behavior is observed. The decrease of the conductivity once Mg enters of ZrO_2_ lattice was related to the increase of the hydroxyl groups evidenced by XPS. It was shown that the electrical conductivity follows the Meyer-Neldel rule (reported here for ZrO_2_ for the first time in the literature), with the parameters σ_oo_, EMN and TMN having values that falls in domains reported for various disordered materials: 1.61 × 10^−7^ (Ω cm)^−1^, 24.62 meV and 285.5 K, respectively. The Meyer-Neldel rule, even largely accepted in the literature to be attributed to the disorder effect in the material, has an origin which was explained for the studied films based on small-polaron hopping model, in the non-adiabatic hopping regime.

## Figures and Tables

**Table 1:** The precursor quantities used to prepare the immersion solutions.

Precursors	The Precursor Quantities (Moles)			
	M0	M1	M2	M3
**ZrCl_4_**	2.45 · 10^−2^	2.43 · 10^−2^	2.41 · 10^−2^	2.40 · 10^−2^
**Mg(NO_3_)_2_**	0	2.45 · 10^−4^	4.90 · 10^−4^	7.41 · 10^−4^

**Table 2:** The binding energy, BE (eV), and the area (A) of the peaks Zr 3d_5/2_, Zr 3d_3/2_, O 1s, Mg 2p_3/2_ and C 1s.

Sample	Zr 3d_5/2_		Zr 3d_3/2_		O 1s		Mg 2p_3/2_		C 1s	
	BE	A	BE	A	BE	A	BE	A	BE	A
M0	182.28	6261	184.61	2950	531.06 532.25	36,177 21,196	-	-	284.58	6389
M1	181.58 182.65	431 8704	183.51 184.97	195 4528	529.55 530.44 532.30	987 30,330 29,343	48.51 49.61 50.73 50.82	86 58 312 84	282.53 * 284.70 286.24 *	426 9072 2980
M2	181.44 182.56	1818 10,556	183.30 184.87	1143 6660	529.62 530.65 532.40	1481 23,696 27,705	48.30 49.63 50.03 50.14	65 97 156 48	281.59 * 284.54 286.87 *	558 10,422 1308
M3	181.27 182.12	849 2286	183.14 184.45	509 1911	530.40 530.86 532.52	9558 11,729 36,874	48.57 49.54 50.14 50.80	104 124 68 22	283.41 * 284.62 285.95 *	3379 7677 647

* satellite.

**Table 3:** The electrical parameters of the studied films.

Sample	E_p_ (meV)	E_h_ (meV)	E_d_ (meV)	H (meV)	γ	E_h_/3 (meV)
M0	365.71	327.55	74.49	34.70	13.3	109.18
M1	250.15	224.47	51.05	31.60	9.13	74.82
M2	256.64	230.11	52.33	31.80	9.36	76.70
M3	202.69	180.99	41.16	29.90	7.36	60.33

## Data Availability

The original contributions presented in the study are included in the article, further inquiries can be directed to the corresponding authors.
